# Efficacy and safety of evocalcet in treatment of secondary hyperparathyroidism in chronic kidney disease on hemodialysis patients: A protocol for a systematic review and meta-analysis

**DOI:** 10.1097/MD.0000000000022566

**Published:** 2020-11-13

**Authors:** Jing Xie, Xueying Li, Yang Chen, Ming Chen, Nan Mao, Junming Fan

**Affiliations:** a Hospital of Chengdu University of Traditional Chinese Medicine; b Department of Nephrology, The First Clinical Medical College; c Chengdu Medical College, Chengdu, PR China.

**Keywords:** calcimimetic agents, chronic kidney disease, evocalcet, secondary hyperparathyroidism, systematic review

## Abstract

**Background:**

Secondary hyperparathyroidism (SHPT) have been associated with poor health outcomes in hemodialysis patients. The cinacalcet has popularized in clinic which has efficacy but more adverse events; the novel oral calcimimetic agents evocalcet has appeared in recent years. However, it is currently unknown whether evocalcet produces more beneficial effects and fewer adverse events in patients with SHPT. The aim of this systematic review is to estimate the safety and efficacy of evocacelt.

**Methods:**

Only randomized controlled trials (RCT) will be included in MEDLINE, EMBASE, the Cochrane Register of Controlled Trials, and PUBMED from July 2010 to July 2020. Two reviewers will screen, select studies, extract data, and assess quality independently. The methodological quality including the risk of bias of the included studies will be evaluated using a modified assessment form, which is based on Cochrane assessment tool. Review Manager 5.3 software will be used for heterogeneity assessment, generating funnel-plots, data synthesis, subgroup analysis, and sensitivity analysis. We will use GRADE system to evaluate the quality of our evidence.

**Results:**

We will provide some more practical and targeted results investigating the effect and safety of evocalcet for SHPT on hemodialysis in the current meta-analysis.

**Conclusion:**

The stronger evidence about evocalcet effect and safety will be provided for clinicians and policymakers.

**Ethics and dissemination:**

Ethical approval will be unnecessary because the data being included in this systematic review come from published literature and there will be no concerns regarding privacy. Findings of this research will be disseminated in a peer-reviewed journal or conference presentations.

**OSF Registration number:**

DOI 10.17605/OSF.IO/N59RB.

## 1 Introduction

Secondary hyperparathyroidism (SHPT) is a major and common complication that develops in chronic kidney disease (CKD) patients undergoing hemodialysis (HD).^[1]^ SHPT is a maladaptive response triggered by hypocalcemia, hyperphosphatemia, and active vitamin D defciency, the increased level of fibroblast growth factor 23 (FGF-23).^[2]^ which in turn causes parathyroid cells to overproduce parathyroid hormone (PTH). Excess levels of PTH are associated with systemic toxicity, known as CKD-mineral and bone disorder (CKD-MBD), which represents the cardiovascular and bone diseases.^[3,4]^ SHPT has typically been treated with the administration of active form of vitamin D to reduce PTH levels^[5,6]^; however, this type of treatment has been associated with elevations in serum calcium and phosphate levels through stimulating gastrointestinal absorption, in addition, which will cause arterial calcifcation^[3]^ and therefore only a few patients were able to achieve the recommended therapeutic targets.^[7]^

Calcimimetics allosterically activate the calcium receptor (CaR) and inhibit the secretion of parathyroid hormone (PTH).^[8]^ Cinacalcet has been approved as the first oral calcimimetic drug for the treatment of SHPT in patients on hemodialysis.^[9]^ Cinacalcet improved the achievement of target serum PTH and Ca levels and helped drastically reduce the number of parathyroidectomies.^[10]^ However, cinacalcet has side effects involving the gastrointestinal tract, such as nausea and vomiting, which makes it difficult to increase the dose and may result in reduced compliance.^[11,12]^

Evocalcet is a novel calcimimetic which has been developed to improve defects of cinacalcet for management of SHPT.^[13]^ Evocalcet acts as an allosteric modulator of CaR, just like cinacalcet.^[14]^ However, its metabolic pathway is different from that of cinacalcet. The metabolism of evocalcet by cytochrome P450 is very low, so evocalcet has higher bioavailability.^[15,16]^ As a result, its pharmacologically effective dose for the inhibition of PTH secretion is lower than that of cinacalcet.^[17,18]^ Evocalcet had less of an effect on the gastrointestinal tract than cinacalcet because of the reduced dose required.^[19]^ Various studys have confirmed that the incidence of gastrointestinal-related adverse events were lower in the evocalcet than in the cinacalcet groups.^[20,21]^ Evocalcet may thus be a potent option for the management of SHPT. In addition, a latest meta-analysis compared effectiveness and adverse events of 3 available calcimimetic agents to treat SHPT in adults with CKD, of whom most were treated with long-term dialysis showed that the cinacalcet ranked worst for nausea and had somewhat lower effectiveness. On contrast, evocalcet has lower effectiveness for achieving target PTH levels while incurring fewer adverse effects.^[22]^

The primary objective is to synthesise the evidence on the effectiveness of evocacelt in improving PTH outcomes for SHPT patients undergoing hemodialysis. Secondary objectives are to estimate the safety and patients-important outcomes, including all-cause mortality, cardiovascular events, cardiovascular mortality, and intermediate outcomes.

## 2 Methods

This review protocol has been registered in the PROSPERO, which is the International Prospective Register of systematic reviews. Its registration number was CRD42020175200.

Cochrane Handbook of Systematic Reviews of Interventions (Version 5.1.0, http://www.cochranehandbook.org) will guide this systematic review. The statement of preferred reporting items for systematic review and meta-analysis protocols^[23]^ and preferred reporting items for systematic reviews and meta analyses (PRISMA)^[24]^ will be used as guidelines for reporting present review protocol and the formal paper that follows.

### 2.1 Eligibility criteria

#### 2.1.1 Types of studies

Only RCTs of evocalcet (KHK7580) will be included, whereas non-RCTs, quasi-RCTs, and any other types of studies will be excluded.

#### 2.1.2 Types of participants

In our study, adult CKD patients on hemodialysis of any severity and elevated serum parathyroid levels, regardless of their age, sex, or race.

#### 2.1.3 Types of interventions

We will include articles comparing treatment groups which received evocalcet. Or of whom were switched from cinacalcet to evocalcet.

#### 2.1.4 Types of outcome assessments

The following biochemical outcomes were considered: the effectiveness laboratory values: intact PTH (iPTH), serum calcium level, serum phosphorus and calcium phosphorus product levels, intact FGF23 concentration, bone turnover markers (bone-specific alkaline phospahatase (BSAP), tartrate-resistant acid phosphatase 5b (TRACP-5b), and total procollagen type 1 intact N-terminal propeptide (P1NP). Patient level outcomes included: all-cause mortality, parathyroidectomy, fractures, hospitalization due to cardiovascular events, cardiovascular mortality. We will also collect all adverse events, hypocalcemia and gastrointestinal-related adverse events such as nausea, vomiting.

#### 2.1.5 Include and exclude criteria

RCTs of evocalcet alone or refer to another calcimimetic agents, parathyroidectomy, placebo, or standard care as treatment of adults with SHPT due to CKD were included. We considered parallel-group and crossover studies of any duration. We excluded studies with primary hyperparathyroidism. And the CKD patients who received peritoneal dialysis, including those with persistent SHPT after kidney transplantation. We excluded studies with a primary objective of optimal dosing or economic evaluation of evocalcet treatment.

#### 2.1.6 Search strategy

The electronic databases MEDLINE, EMBASE and the Cochrane Central Databases will be searched using standard controlled vocabulary (MeSH or EMTREE), text words, and keywords. It is considered that which was prescribed in Japan widely. We will search for conference proceedings and articles from May 2010 to June 2020 using the EBM and abstracts presented in recently (The Japanese Society for Dialysis Therapy and The Japanese Pharmacological Society). Please refer to Table 1 for the full search strategy.

**Table 1:** Search strategy for the MEDLINE and cochrane central register of controlled trials databases.

	MEDLINE	
Set	History	comments
1	(((kidney^∗^ or nephro^∗^ or renal or home or peritoneal or intermittent or chronic or extracorporeal orambulatory) adj2 (haemodialys^∗^ or hemodialys^∗^ or dialys^∗^)) or hemorenodialysis or hemodialyse orCAPD). ti, ab.	Dialysis textword search Terms
2	renal dialysis/ or hemodialysis, home/ or peritoneal dialysis/ or peritoneal dialysis, continuousambulatory/	Dialysis subject Terms
3	renal insufficiency, chronic/ or kidney failure, chronic/	Chronic kidney disease subject terms
4	(((chronic or “end-stage” or “end stage”) adj3 (kidney^∗^ or renal or nephro^∗^) adj3 (insufficien^∗^ ordisease^∗^)) or esrd). ti, ab.	Chronic kidney disease textword terms
5	frasier syndrome/ or (“frasier^∗^ syndrome^∗^” or (frasier^∗^ adj2 syndrome^∗^)). mp	Syndrome subject and textword terms
6	renalosteodystrophy/ or ((renal or kidney^∗^ or nephro^∗^) adj2 (osteodystroph^∗^ or ricket^∗^)). mp.	Chronic kidney disease subject or textwordTerms
7	azotemia/ or azotemi^∗^. mp	Azotemia subject or textword Terms
8	uremia/ or uremi^∗^. mp.	Uremia disease subject or textword Terms
9	or/1–8/NOT (pediatic or chriden)	Kidney disease terms
10	Calcimimetic Agents/ or (Calcimimetic^∗^ or ORKEDIA or evocalcet or “khk7580” or khk7580). mp	Evocalcet subject and textword searchTerms
11	9 and 10	Base clinical set
12	controlled clinical trial.pt. or controlled clinical trials as topic/ or meta analysis.pt. ormeta analysis astopic/ or multicentre study.pt. or multicenter studies as topic/ or randomized controlled trial.pt. orrandomized controlled trials as topic/ or pragmatic clinical trial.pt. or Pragmatic Clinical Trials asTopic/ or ((preference or practical or pragmatic or “real world” or naturalistic) adj5 trial^∗^).ti, ab. orComparative Effectiveness Research/ or ((comparative adj2 effectiveness) or (CER adj5 (research^∗^ ormethod^∗^ or framework^∗^ or compari^∗^ or statement^∗^))). ti, ab. or ((singl: or doubl: or tripl: or trebl:) and(mask: or blind:)). ti, ab. or ((random: adj5 trial:) or rct or rcts). ti, ab.	Therapy Study design methodologies
13	11 and 12	FINAL Results
	EBM Reviews - Cochrane Central Register of Controlled Trials	
1	((kidney^∗^ or nephro^∗^ or renal or home or peritoneal) adj2 (haemodialys^∗^ or hemodialys^∗^ ordialys^∗^)). ti, ab. or renal dialysis/ or hemodialysis, home/ or peritoneal dialysis/ or peritoneal dialysis,continuous ambulatory/ or renal insufficiency, chronic/ or kidney failure, chronic/ or (((chronic or“end-stage” or “end stage”) adj3 (kidney^∗^ or renal or nephro^∗^) adj3 (insufficien^∗^ or disease^∗^)) oresrd). ti, ab. or frasier syndrome/ or (“frasier^∗^ syndrome^∗^” or (frasier^∗^ adj2 syndrome^∗^)). mp. or renalosteodystrophy/ or ((renal or kidney^∗^ or nephro^∗^) adj2 (osteodystroph^∗^ or ricket^∗^)). mp. orazotemia/ or azotemi^∗^.mp. or uremia/ or uremi^∗^.mp. or (((kidney^∗^ or nephro^∗^ or renal or home orperitoneal or intermittent or chronic or extracorporeal or ambulatory) adj2 (haemodialys^∗^ orhemodialys^∗^ or dialys^∗^)) or hemorenodialysis or hemodialyse or CAPD). ti, ab. or kidney failure/ orchronic kidney failure/ or frasier syndrome/ or renal osteodystrophy/ or uremia/ or (((chronic or“end-stage” or “end stage”) adj3 (kidney^∗^ or renal or nephro^∗^) adj3 (insufficien^∗^ or disease^∗^)) oresrd). ti, ab. or kidney failure/ or chronic kidney failure/ or frasier syndrome/ or (“frasier^∗^ syndrome^∗^”or (frasier^∗^ adj2 syndrome^∗^)). mp. or renal osteodystrophy/ or ((renal or kidney^∗^ or nephro^∗^) adj2(osteodystroph^∗^ or ricket^∗^)).mp. or azotemia/ or azotemi^∗^.mp. or uremia/ or uremi^∗^.mp.	Dialysis, chronic kidney diseases Subject ortextword search Terms
2	Calcimimetic Agent/ or Evocalcet/ or Calcimimetic Agents/ or (Calcimimetic^∗^ or ORKEDIA or evocalcet or “khk7580” or khk7580).mp.	Cinacalcet subject and textword searchTerms
3	(1 and 2)NOT (pediatic or chriden)	Base clinical set

### 2.2 Data collection and analysis

#### 2.2.1 Selection of studies

Teams of 2 investigators independently screened each unique title and abstract identified in our literature search. If either reviewer identified a citation as potentially relevant, we obtained the full text of the article. Two reviewers independently determined the eligibility of all studies that underwent full text evaluation.

**Figure 1:**
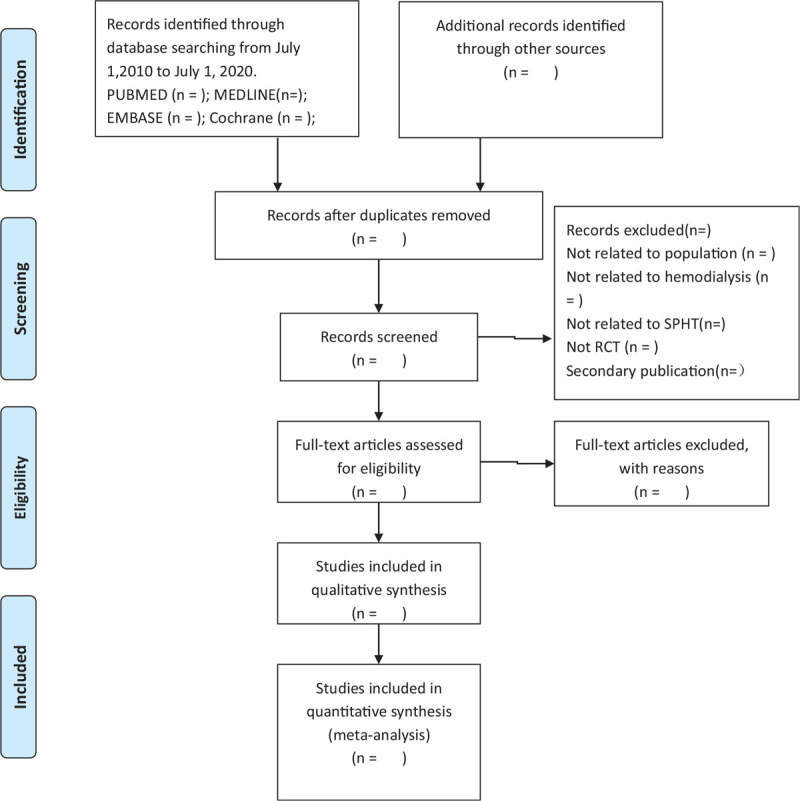
Flow diagram of study selection. RCT = randomized controlled trial.

We measured the inter-rater agreement for full text eligibility and assessment of risk of bias using the kappa statistic.^[25]^ Values of kappa between 0.40 and 0.59 reflect fair agreement, between 0.60 and 0.74 reflect good agreement and ≥0.75 reflects excellent agreement. Disagreements were resolved through discussion between reviewers or through adjudication with a third party if necessary. Details of the entire selection process are shown in a PRISMA flow chart^[26]^ (Fig. 1).

#### 2.2.2 Data and information extraction

Using a standardized data collection form, 2 investigators abstracted the following information from each study: author, date of publication, eligibility criteria, summary of baseline characteristics of the participants, number of participants in each arm at study onset and completion, duration of the trial and treatment effects, including effectiveness and safety. We resolved disagreements by discussion.

#### 2.2.3 Dealing with missing data

We employed a complete-case analysis for our primary analysis and planned to conduct sensitivity analyses to address the robustness of our findings with respect to missing data. We planned to use plausible worst-case scenario for missing trial-level data.

#### 2.2.4 Appraisal of study quality

If there are eligible randomized controlled trials, quality will be evaluated using the Cochrane Risk Assessment Tool. Studies will be assessed on randomization, generation of allocation sequence, allocation concealment, blinding and follow-up. The quality of evidence across studies will be assessed for each outcome using the Grading of Recommendations, Assessment, Development and Evaluation (GRADE) approach. GRADE considers the risk of bias, consistency of results across studies, precision of the overall estimate across studies, magnitude of effect and importance of the outcome.^[27]^ The quality of evidence will be rated as high, moderate, low or very low for each outcome.

#### 2.2.5 Assessment of reporting bias

If there are no <10 studies available for quantitative analysis, we will generate funnel plots to assess reported bias. For continuous variables, the Egger test will also be adopted to check the asymmetry of funnel plots. However, even if the test does not provide evidence of funnel plot asymmetry, reporting bias (including publication bias) cannot be excluded due to the relatively low testing capacity. Asymmetric funnel plots are generally considered to have publication bias, which is a type of reporting bias, but it also implies that there may be other causes, such as differences in methodological quality or true heterogeneity of intervntion effects. We will analyze the possible reasons and give a reasonable explanation for the asymmetric funnel plot.

#### 2.2.6 Assessment of heterogeneity

We formally assessed heterogeneity using Cochrans Q test, Chi-Squared test of homogeneity and the *I*^2^ statistic for which 0% to 40% may be unimportant heterogeneity, 30% to 60% moderate, 50% to 90% substantial, and 75% to 100% considerable heterogeneity.^[28]^ For cardiovascular mortality, we employed random effects meta-regression and included mean age, the mean baseline serum PTH concentration, trial duration in our univariate linear models. For all-cause mortality, we employed random effects meta-regression and included trial duration in our univariate linear models.

#### 2.2.7 Data synthesis and statistical analysis

We used contrast-level summary data to perform pairwise meta-analysis based on normal models. We reported descriptive statistics as proportions for categorical variables and mean or medians for continuous variables. We calculated pooled risk ratios (RRs) and the associated 95% CI for each outcome using random effects models by applying the maximum likelihood method. We also calculated absolute effects and the associated 95% CIs by multiplying pooled RRs and 95% CI by the control rate of outcomes from the RCT at low risk of bias and with the largest sample size.^[29]^ Quantitative data synthesis will be carried out by Review Manager software (Revman5.3, available from the Cochrane Web site: http://tech.cochrane.org/Revman).

#### 2.2.8 Subgroup analysis

If heterogeneity is evaluated as significant, we will perform a subgroup analysis to explore the possible causes of heterogeneity according to the difference in participant characteristics, interventions, controls, and outcome measures.

#### 2.2.9 Sensitivity analysis

We plan to conduct sensitivity analysis by excluding combined studies one by one to observe whether there is signifificant change in the comprehensive results. Signifificant changes are reflected in studies that are suffificient to affect the overall synthesis results, so it is necessary to reevaluate them and make a careful decision whether to merge or not. We must give a reasonable reason before we make a decision. If there is no signifificant change, we can assume that our overall results are firm.

#### 2.2.10 Ethics and dissemination

Ethical approval will be unnecessary because the data included in this systematic review come from published literature and there will be no concerns regarding privacy. Findings of this research will be disseminated in a peer-reviewed journal or conference presentations.

## 3 Discussion

We expect to provide an objective assessment of effectiveness and safety of evocalcet in patients with SHPT on hemodialysis. We will examine the impact of evocalcet on patient important outcomes. The data on the bone turnover markers will be concluded. However, this is believed to be due to the influence of pre-treatment with cinacalcet. The long term adverse events assessment of evocalcet and patient important outcomes available in the literature. We will disseminate our results in local meetings and in a peer-reviewed publication.

The methods of our proposed review are state of the art, including explicit eligibility criteria, a comprehensive search, independent duplicate assessment of eligibility, and the use of the GRADE approach to assessing quality of evidence of effect including independent duplicate assessment of risk of bias, precision, consistency, directness, and publication bias. Our protocol represents a model for systematic review methods. Our results are likely to be limited by limitations in the primary studies. One limitation of this review is that we will only search Chinese and English databases, possibly missing some articles published using other language. This study was also limited by not being placebo controlled, having a small number of patients, the possibility of attrition bias, adopting only Japaneses Society for Dialysis Therapy iPTH level standord and including only Japanese patients, which limits the generalizability of the results.

## Author contributions

**Conceptualization:** Jing Xie, Junming Fan.

**Data curation:** Jing Xie, Xueying Li.

**Funding acquisition:** Nan Mao, Junming Fan.

**Investigation:** Xueying Li, Ming Chen.

**Methodology:** Xueying Li, Yang Chen.

**Software:** Jing Xie, Xueying Li, Yang Chen.

**Supervision:** Nan Mao, Junming Fan.

**Writing – original draft:** Jing Xie.

**Writing – review & editing:** Jing Xie.
